# A standardized microarray assay for the independent gene expression markers in AML: EVI1 and BAALC

**DOI:** 10.1186/2162-3619-2-7

**Published:** 2013-03-06

**Authors:** Jaap Brand, Martin H van Vliet, Leonie de Best, Peter JM Valk, Henk E Viëtor, Bob Löwenberg, Erik H van Beers

**Affiliations:** 1 Skyline Diagnostics BV, Rotterdam Science Tower, Marconistraat 16, 3029 AK, Rotterdam, The Netherlands; 2 Department of Hematology, Erasmus University Medical Center, Rotterdam, The Netherlands

**Keywords:** AML, Acute myeloid leukemia, *BAALC*, Brain and acute leukemia cytoplasmic, *EVI1*, Ecotropic viral integration site 1, Intermediate cytogenetic risk, Prognosis, OS, Overall survival

## Abstract

High levels of *BAALC*, *ERG*, *EVI1* and *MN1* expression have been associated with shorter overall survival in AML but standardized and clinically validated assays are lacking. We have therefore developed and optimized an assay for standardized detection of these prognostic genes for patients with intermediate cytogenetic risk AML. In a training set of 147 intermediate cytogenetic risk cases we performed cross validations at 5 percentile steps of expression level and observed a bimodal significance profile for *BAALC* expression level and unimodal significance profiles for *ERG* and *MN1* levels with no statistically significant cutoff points near the median expression level of *BAALC*, *ERG* or *MN1*. Of the possible cutoff points for expression levels of *BAALC*, *ERG* and *MN1*, just the 30th and 75th percentile of *BAALC* expression level and the 30th percentile of *MN1* expression level cutoff points showed clinical significance. Of these only the 30th percentile of *BAALC* expression level reproduced in an independent verification (extended training) data set of 242 cytogenetically normal AML cases and successfully validated in an external cohort of 215 intermediate cytogenetic risk AML cases. Finally, we show independent prognostic value for high *EVI1* and low *BAALC* in multivariate analysis with other clinically relevant molecular AML markers. We have developed a highly standardized molecular assay for the independent gene expression markers *EVI1* and *BAALC*.

## Background

Overexpressions of *EVI1*, *BAALC*, *ERG*, and *MN1* have been reported to be prognostically relevant in AML [1-9]. For instance, the prognostic value of *EVI1* overexpression was discovered and reproduced in intermediate cytogenetic risk AML [4,9-13], while the prognostic value of *BAALC*, *ERG* and *MN1* mRNA values were demonstrated in normal karyotype AML [1,6,8]. These studies selected univariate cutoff points for *BAALC*, *ERG*, and *MN1* continuous expression levels based on cohort quartiles, while the *EVI1* expression cutoff point was chosen to discriminate between undetectable or low levels versus high expression levels. Translation to the clinic has been proposed [14-20] but lack of standardized assays has hampered their broad implementation. We have developed a prognostic assay on a custom gene expression array that detects *EVI1* overexpression and low *BAALC* expression levels in individual AML patients as part of a multiplex genetic array that also detects AML with t(8;21), t(15;17), inv(16)/t(16;16), *NPM1* mutations, and *CEBPA* double mutations with high accuracy (sensitivity and specificity > 95%).

## Results and discussion

### OS prognostic assay for BAALC, ERG, and MN1

**Figure 1:**
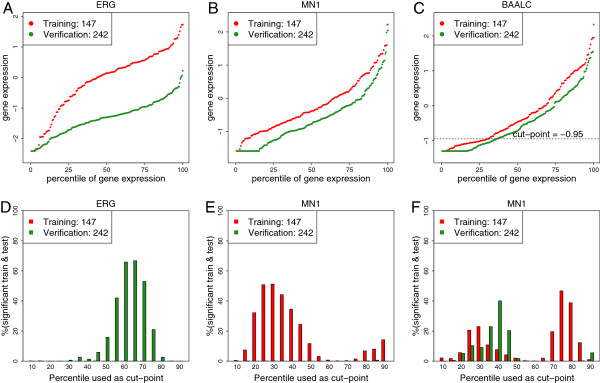
**Expression distribution for *ERG*, *MN1 *and *BAALC *in intermediate cytogenetic risk AML.** Figure **A**-**C**; x-axes shows cases sorted by expression in training (red) or verification (green) cohorts, while the y-axis shows standardized expression values. Figure **D**-**F**; x axis shows all seventeen 5-percentile intervals between 10 and 90. The y-axis indicates the significant fraction (%) of 1000 folds cross-validation draws in the training cohort (red bars) or verification cohort (green bars) for *ERG*, *MN1* and *BAALC*, respectively. Significance is defined by log rank p-value < 0.05.

*BAALC*, *ERG* and *MN1* gene expression levels were determined in a standardized assay suitable for single case analysis (see Methods) in a training set, an independent verification (extended training) set and one independent validation set of AML patients. Distributions of *ERG* mRNA levels on average were higher in the training cohort as compared with the verification cohort (Figure 1A) while *MN1* and *BAALC* expression levels were similar (Figure 1B and C). Results of 1000-fold cross-validations (CV) in the training and verification cohorts for *BAALC*, *ERG*, and *MN1* expression levels (Figure 1D-F). For *BAALC* expression levels there are two local optima in the training cohort at the 30^th^ percentile cutoff point and 75^th^ percentile cutoff points with 23% and 47% significant folds (y-axis) with a log rank for OS p < 0.05. At the 25^th^, 30^th^ and 35^th^ percentile there are 10%, 9% and 23% of the 1000 random cohort splits in the validation cohort (Figure 1D, green bars). Clearly only the 30^th^ percentile *BAALC* cutoff point is supported by the verification cohort and thus chosen for lock-down and further validation.

No significant cutoff point for *ERG* expression levels were found in the training cohort at any of 17 expression cutoff points analyzed (Figure 1E), because the percentage (y-axis in Figure 1E) of the 1000 random cohort splits was < 1% for every cutoff point. Therefore, due to ambiguous training and verification results, *ERG* expression levels were not considered for validation. For *MN1* mRNA expression levels (Figure 1F) there is an optimum at the 30^th^ percentile in the training cohort corresponding with a normalized expression value −0.76 and achieving 51% significant cross validation splits. Although, this cutoff point could not be reproduced in the independent verification cohort, it was assessed for further validation on an independent cohort. The prognostic value of both *ERG* and *MN1* expression levels for overall survival is inconsistent between training and verification cohorts (Figure 1).

### Finding a clinically relevant cutoff point for EVI1 expression

**Figure 2:**
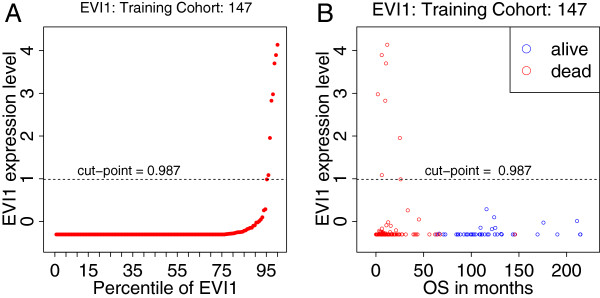
**Plot of *EVI1 *expression versus the percentile of *EVI1 *(A) and of *EVI1 *gene expression versus overall survival (OS) in months (B) both for the training cohort.**

The distribution of *EVI1* mRNA expression levels in the training cohort is extremely skewed as can be seen in Figure 2A. Figure 2A also shows the cutoff point of 0.987, which was derived by maximizing the logrank test statistic (see Statistical analysis). All cases with a high *EVI1* expression level (above the cutoff point) have a short survival and died (Figure 2B, red circle) while the cases with a low *EVI1* expression level (below the cutoff point) have much longer survival.

### Cutoff point validation

**Figure 3:**
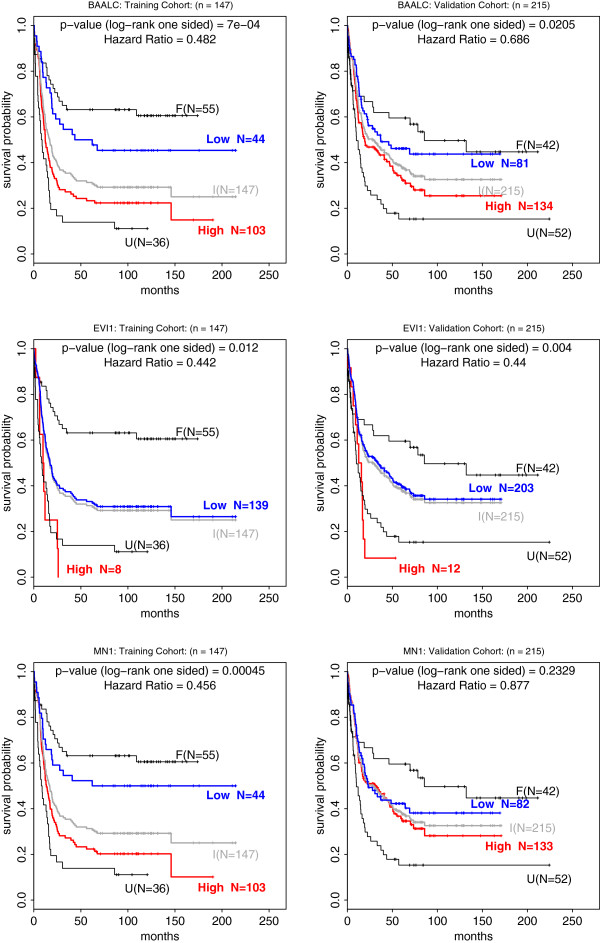
**Kaplan-Meier analysis of overall survival (OS) for *BAALC*, *EVI1 and MN1 *in training (left) and validation cohorts (right).** Individual KM strata are color coded to depict favorable (F), intermediate (I), unfavorable (U) cytogenetic risk. The intermediate group is plotted in grey because it was re-stratified into low expressors (blue) and high expressors (red). cutoff points used in this validation study are BAALC expression −0.95 (derived from the 30^th^ percentile in the training), EVI1 expression 0.987 (derived from a single short survival case in the training cohort), MN1 expression −0.76 (derived from the 30^th^ percentile in the training).

The prognostic significance for OS between *BAALC* low-expressers and high-expressers in the training (left) cohort and validation (right) cohort (Table 1 and Figure 3) (HR 0.482, p-val 7 × 10^-4^ and HR 0.686, p-val = .0205) and for low *EVI1* expression (HR 0.442, p-val .012 and HR 0.44, p-val .004) and therefore both pass the validation. However, *MN1* gene expression levels is only statistically significant for the training cohort (HR 0.456, p-val 0.00045) but not for the validation cohort (HR 0.877, p-val 0.2329) and thus will not be considered further. Since for *ERG* expression levels no significant cutoff point was identified in the training cohort (Figure 1) it was not included in the validation study.

**Table 1:** Hazard ratio and logrank (p-value) for evaluated cut points in training, verification and validation datasets

**Gene name**	**Dataset**	**AMLprofiler**	**25**^**th **^**percentile**	**50**^**th **^**percentile**	**75**^**th **^**percentile**
*BAALC*	Training	**.48 (.001)**	**.44 (< .001)**	**.63 (.01)**	**.43(< .001)**
	Verification *)	**.60 (.002)**	**.55 (.001)**	**.66(.005)**	**.65 (.007)**
	Validation	**.69 (.021)**	.72 (.066)	.88 (.24)	.93 (.345)
*ERG*	Training	No significant cutoff point	1.41 (.066)	1.37 (.051)	**1.45 (.048)**
	Verification *)	ND	**1.41 (.037)**	**1.56 (.003)**	**1.72 (.001)**
	Validation	ND	**1.54 (.02)**	1.12 (.248)	1.28 (.099)
*MN1*	Training	**2.19 (< .001)**	**2.56 (< .001)**	**1.79 (.002)**	**1.79 (.003)**
	Verification *)	**1.31 (.049)**	1.12 (.26)	**1.32 (.049)**	**1.39 (.035)**
	Validation	1.14 (.2329)	1.37 (.068)	1.19 (.167)	1.32 (.081)

OS logrank p < 0.05 indicated in bold. For *ERG* no cut point was identified in the training cohort.

*) this data set contains only normal karyotype cases.

ND, not done because lack of significant cutoff point in training.

### Cutoff point in relation to event free survival

*Low BAALC* and high *EVI1* were also prognostic for EFS in the training and validation cohorts *BAALC* (training p = 0.0038; validation p = 0.0105 by the logrank test) and *EVI1* (training p = 0.0164; validation p = 0.00125 by the logrank test), respectively.

### NPM1, CEBPA and FLT3 mutation frequencies in BAALC, EVI1 expression subgroups

We examined the distribution of AML mutations *NPM1*, *CEBPAdm* and *FLT3*-*ITD* among low *BAALC* and high *EVI1* expression AML, respectively (Tables 2 and 3). Low *BAALC* expression cases had significantly more *NPM1* mutations (49/85) compared with high *BAALC* expressors (36/85) (Fisher’s exact, p < 0.0001). All 10 *CEBPA* double mutants were present in high *BAALC* expressors and therefore significantly enriched (Fisher’s exact, p = 0.0146). *FLT3*-ITD mutant frequency did not differ between low (25/81) or high (54/134) *BAALC* expressors (Fisher’s exact, p = 0.148).

**Table 2:** **Mutations at diagnosis stratified for *BAALC *expression levels in the validation cohort**

**Variable**	**Total n = 215**	**Low *BAALC *n =81**	**High *BAALC *n = 134**	**Fisher’s exact test p-value**
*NPM1*-*ABD*	85	49	36	**<0.0001**
*CEBPA*-dm	10	0	10	**0.0146**
*FLT3*-*ITD*	79	25	54	0.148

**Table 3:** **Mutations at diagnosis stratified for *EVI1 *expression levels in the validation cohort**

**Variable**	**Total n = 215, (%)**	**Low *EVI1 *n =203, (%)**	**High *EVI1 *n =12, (%)**	**Fisher’s exact test p-value**
*NPM1*-*ABD*	85	85	0	**0.0039**
*CEBPA*-dm	10	10	0	1
*FLT3*-*ITD*	79	77	2	0.218

*NPM1* mutations were enriched (85/203) in low *EVI1* compared with none in 12 high *EVI1* (Table 3, p = 0.0039). *CEBPA* double mutant frequency did not differ between low *EVI1* expressors (10/203) or high *EVI1* expressors (0/12) (Fisher’s exact, p = 1). And finally, *FLT3* mutation frequency did not significantly differ between low and high *EVI1* expressors (Table 3, p = 0.218).

### Multivariate analysis

The prognostic value of *BAALC* and *EVI1* expression levels was further tested in a multivariate Cox-Proportional Hazard analysis in the validation cohort adjusting for potential confounding covariates including the mutation markers *CEPBA* double mutations, *NPM1* mutations, *FLT3*-ITD, age in years, gender, White Blood Cell count, percent of blast cells in bone marrow and platelet count at diagnosis (Table 4). When adjusting for these variables, *EVI1* overexpression proved an independent significant prognostic factor (p = 0.019; HR = 2.21; Table 4), but *BAALC* expression levels not. Therefore we have also analyzed the validation cohort after excluding all n = 12 *EVI1* overexpression cases and demonstrate that low *BAALC* expression level remains an independent prognostic factor (p = 0.035; HR =0.62; Table 5) when evaluated in all (n = 203) low *EVI1* cases.

**Table 4:** Multivariate analysis in the validation cohort for OS using Cox Proportional Hazard model

**Variable**	**p-value**	**HR**	**95% CI**
*BAALC*	0.1	1.42	0.93–2.15
*EVI1*	**0.019**	2.21	1.14–4.27
*CEBPA*-*dm*	0.052	0.36	0.13–1.01
*NPM1*-*ABD*	0.21	0.76	0.50–1.17
*FLT3*-*ITD*	**0.017**	1.64	1.09–2.46
age in years (continuous variable, in yrs)	**0.021**	1.02	1.00–1.03
gender	0.76	1.06	0.73–1.55
White blood Cell Count at diagnosis [×10^9/l]	0.17	1.00	1.00–1.00
Percentage of blast cells in bone marrow	0.45	1.00	0.99–1.00
Platelets at diagnosis [×10^9/l]	0.24	1.00	1.00–1.00

Gene expression, gene mutation and gender variables are binary, while age, WBC, % blast, and platelets are continuous variables.

**Table 5:** **Multivariate analysis in the validation cohort excluding high *EVI1 *cases**

**Variable**	**p-value**	**HR**	**95% CI**
*BAALC*-*low*	**0.035**	1.56	1.03–2.54
*CEBPA*-*dm*	**0.047**	0.35	0.12–0.98
*NPM1*-*ABD*	0.36	0.82	0.53–1.26
*FLT3*-*ITD*	**0.038**	1.56	1.02–2.39
age in years	**0.012**	1.02	1.00–1.04
gender	0.74	1.07	0.72–1.58
White blood Cell Count at diagnosis [×10^9/l]	0.14	1.00	1.00–1.00
Percentage of blast cells in bone marrow	0.2	0.99	0.99–1.00
Platelets at diagnosis [×10^9/l]	0.28	1.00	1.00–1.00

Gene expression, gene mutation and gender variables are binary, while age, WBC, % blast, and platelets are continuous variables.

## Conclusions

We have developed a standardized assay for *BAALC* and *EVI1* gene expression markers with prognostic value for patients with AML. We trained an assay on a well-characterized cohort of intermediate cytogenetic risk AML cases and determined cutoff points for the gene expression markers *BAALC* and *EVI1*. Similar to previous studies the cutoff point for *EVI1* overexpression was selected and validated to predict for worse OS in AML patients. Low *BAALC* was trained as those cases with the lowest 30^th^ percentile *BAALC* expression level and found to predict for significantly worse OS in an independent cohort of intermediate cytogenetic risk cases (Table 1). Both *EVI1* overexpression and low *BAALC* expression levels were significantly associated with clinical outcome as shown by multivariate analysis, including other molecular markers such as *NPM1*, *FLT3* and *CEBPA* gene aberrations. *Two o*ther prognostic gene expression markers, *evaluated in this study*, *MN1* and *ERG* were found not significantly prognostic in either training or validation cohorts and therefore not added to the AMLprofiler assay. We successfully standardized and validated OS prognostic assays for low *BAALC* and high *EVI1* expression levels in AML that we integrated into an *in vitro* diagnostic platform for clinical use that simultaneously detects t(8;21), t(15;17), inv(16), t(16;16), *NPM1*, and *CEBPA* double mutations.

## Methods

### Patients and treatment

This study used three datasets, a training cohort, a verification cohort and a validation cohort. The training cohort consisted of 147 intermediate cytogenetic risk AML cases, the validation cohort of 215 intermediate cytogenetic risk cases from the HOVON collaborative treatment group (http://www.hovon.nl; studies HOVON-4, -29, -32, -42 and -43). All subjects provided written informed consent in accordance with the Declaration of Helsinki. This research has been approved by the Medical Ethical Committee of the Erasmus University Medical Center. The verification cohort consisted of 242 cytogenetically normal AML cases and was publically available ([21], GSE12417).

### Measurements of BAALC, EVI1, ERG and MN1 expression

Training and validation expression levels are measured in RNA extracted from ficoll purified blast cells from diagnostic BM and PB samples as previously described [22]. The stored hybridization cocktails have been re-hybridized to the AMLprofiler custom GeneChip which has 995 probe sets that are a subset of the Affymetrix U133Plus2.0 GeneChip (n = 505 cases, GSE42194). We had previously validated 10 re-hybridizations of cocktails including the freeze-thaw cycles and could not show impact on mRNA quantification (data not shown). Probe set intensity data for the external cohort are obtained from the Gene Expression Omnibus (http://www.ncbi.nlm.nih.gov/geo/; accession GSE12417) and copied from U133Plus2.0 format into the corresponding 995 probe set coordinates of a dummy AMLprofiler to guarantee standardized data analysis including MAS5.0 summarization, chip normalization and Geometric Mean Centering per probe set (gene). Next, the expression level of *BAALC* is calculated as the average of probe sets 218899_s_at and *222780*_s_at after mean variance normalization. The expression level of *ERG* is the average of probe sets 241926_s_at and 213541_s_at after mean variance normalization and the expression level of *EVI1* is the average of probe sets 221884_at and 226420_at after mean variance normalization. The expression level of *MN1* is the value of the probe set 205330_at.

### Cutoff point development

Cutoff points for *BAALC*, *ERG* and *MN1* expression levels were developed using a 147 case training cohort of intermediate cytogenetic risk AML as well as a 242 case normal karyotype AML cohort with overall survival (OS) information. Figure 1 then served to derive optimal cutoff points. It shows results of 1000 random repetitions of cross-validation in training and verification cohorts stratifying between 10–90^th^ percentile expression levels in steps of 5%. In each repetition the particular cohort is randomly split into 50% train and 50% test cases. The results of the test cases are used to calculate the logrank for OS between high and low cases. The number of significant p-values (logrank p < 0.05) during 1000 repetitions is plotted on the y-axis. For each gene a cutoff point was chosen from the optimum significance in the training set. As two peaks were observed for *BAALC* the verification dataset (Figure 1, green bars) guided the choice for the peak at the 30^th^ percentile (Figure 1). For *MN1* the 30^th^ percentile was the only optimum and no clear significant peak was seen in the verification data. For ERG, there was no optimum in the training data, but only in the verification data. The cutoff points for *BAALC* and *MN1* were then translated from percentile value to their corresponding expression levels. For *BAALC* (−0.95) and for *MN1* (−0.76). These expression levels were locked-down for external significance testing in the validation data set. The cutoff point selection was different for *EVI1*. Because the expression distribution is skewed towards very low or no expression with just a few percent of cases with high expression (Figure 2) the cutoff value was chosen at 0.987 such that 12 cases are annotated as having high *EVI1* expression with significantly shorter OS.

### Statistical analysis

Standardized methods for prognostic stratification of AML patients with intermediate cytogenetic risk based on the genes *BAALC*, *ERG*, *MN1* and *EVI1* are established by selecting an appropriate cutoff point for each gene that classifies patients into low- or high expressers. Because of the extremely skewed distribution of *EVI1* expression level, the above CV procedure does not have enough power to yield a meaningful result for *EVI1* overexpression.

### Assay validation

The cutoff points for the genes *BAALC* and *EVI1* derived from the training cohort were validated by means of Kaplan-Meier analysis of low expressers (below cutoff point) versus high expressers (above cutoff point). A gene and cutoff point passes the validation if the one-sided p-value with respect to difference in OS between low expressers and high expressers according to the log-rank test is statistically significant, i.e., p ≤ 0.05. A one-sided p-value is justified because for each of the four genes there is prior knowledge that a higher expression predicts for worse OS prognosis.

## Abbreviations

AML: Acute myeloid leukemia; BAALC: Brain and acute leukemia cytoplasmic; BM: Bone marrow; CEBPAdm: CCAAT/enhancer binding protein alpha; CV: Cross validation; ERG: *ETS*-related gene; EVI1: Ecotropic Viral Integration 1; FLT3: *FMS*-like tyrosine kinase; HR: Hazard ratio; MN1: Meningioma (disrupted in balanced translocation) 1; NPM: Nucleophosmin; OS: Overall survival; PB: Peripheral blood; WBC: White blood cells.

## Competing interests

JB, MHV, LB, PJMV, BL, HV, and EHB report equity in Skyline Diagnostics.

## Authors’ contributions

JB analyzed the data, and wrote the article; MHV analyzed data, co-designed the cutoff points and reviewed the manuscript; LB co-designed the cutoff points and reviewed the manuscript, PJMV, and BL provided patient samples and reviewed the manuscript, HEV designed the study and reviewed the manuscript, EHB designed the study, analyzed the data, wrote the manuscript and gave final approval of the submitted manuscript. All authors have read and approved the final manuscript.
